# Supplementary material for: Minimally invasive pyeloplasty versus open pyeloplasty for ureteropelvic junction obstruction in infants: a systematic review and meta-analysis
Source: PeerJ. 2023 Nov 20;11:e16468. doi: 10.7717/peerj.16468 (PMC10666611; doi:10.7717/peerj.16468)
Supplement: Supplemental Information 2 [file peerj-11-16468-s002.docx]

**Identification of studies via databases and registers**

Records removed *before screening* (n=1469):

Duplicate records removed (n=635)

Records marked as ineligible by automation tools (n=293)

Records removed for other reasons (n=541)

Not comparative study (n=125)

Not relevant intervention (n=137)

Not relevant patients (n=279)vant patients(n=64)

Records identified from(n=1941):

Databases

(PubMed:n=283;Cochrane:n=217; Embase:n=239; CNKI:n=389; Wangfang: n=412; VIP: n=401)

**Identification**

Records excluded(n=388):

Not original papers (n=183)

Not comparative records (n=126)

Not relevant patients (n=79)

Records screened

(n = 472)

Reports sought for retrieval

(n =84)

Reports not retrieved

(n = 39)

**Screening**

Reports excluded (n=34):

Not targeted comparisons (n=17)

Not relevant outcomes (n=9)

Not complete data (n=8)

Reports assessed for eligibility

(n =45)

Studies included in review

(n = 11)

**Included**

*Consider, if feasible to do so, reporting the number of records identified from each database or register searched (rather than the total number across all databases/registers).

**If automation tools were used, indicate how many records were excluded by a human and how many were excluded by automation tools.

*From:*  Page MJ, McKenzie JE, Bossuyt PM, Boutron I, Hoffmann TC, Mulrow CD, et al. The PRISMA 2020 statement: an updated guideline for reporting systematic reviews. BMJ 2021;372:n71. doi: 10.1136/bmj.n71

For more information, visit: <http://www.prisma-statement.org/>
