# Supplementary figures and images for: Minimally invasive pyeloplasty versus open pyeloplasty for ureteropelvic junction obstruction in infants: a systematic review and meta-analysis
Source: PeerJ. 2023 Nov 20;11:e16468. doi: 10.7717/peerj.16468 (PMC10666611; doi:10.7717/peerj.16468)

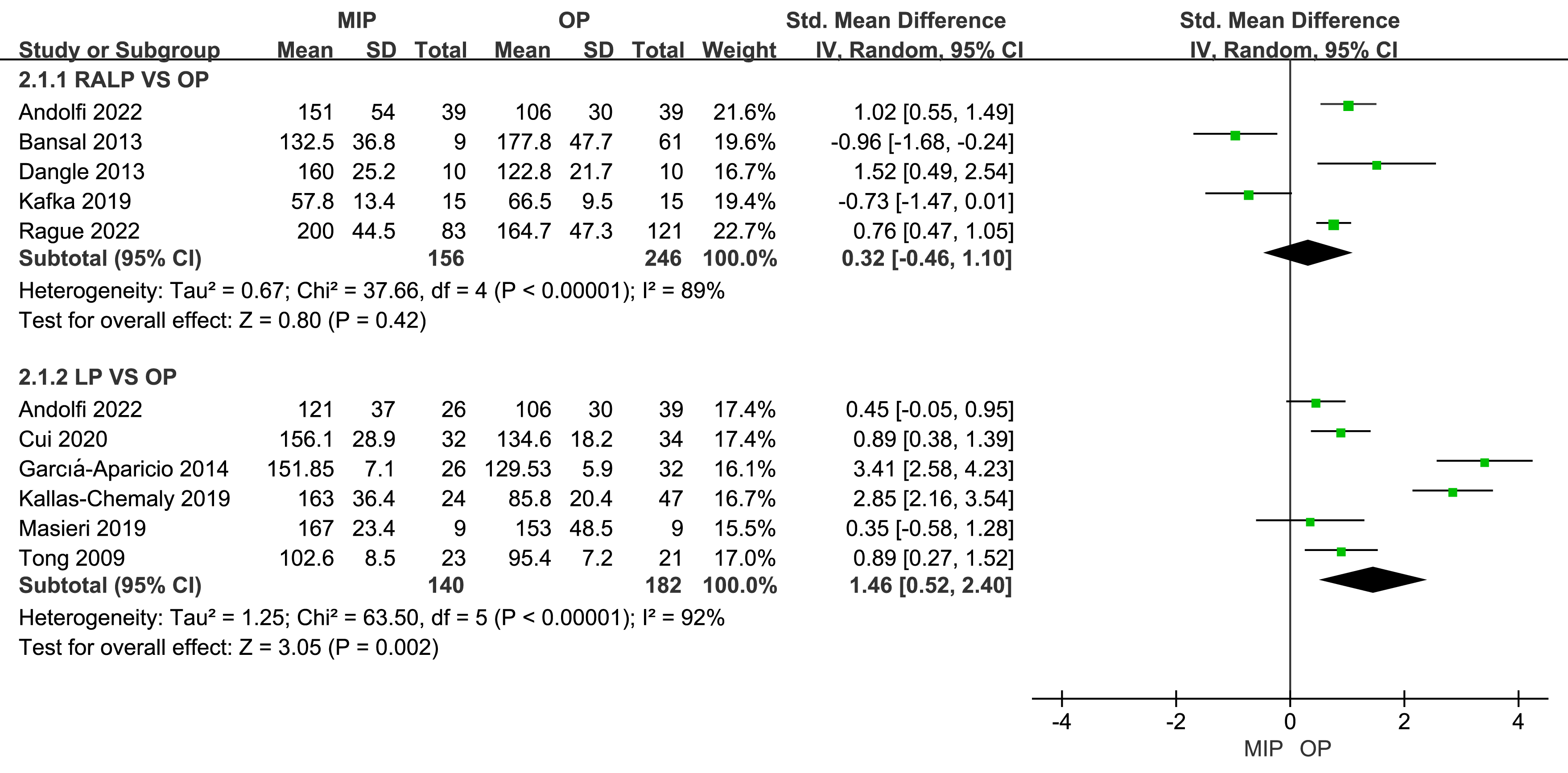

Supplement: Figure S1 [file peerj-11-16468-s004.png]

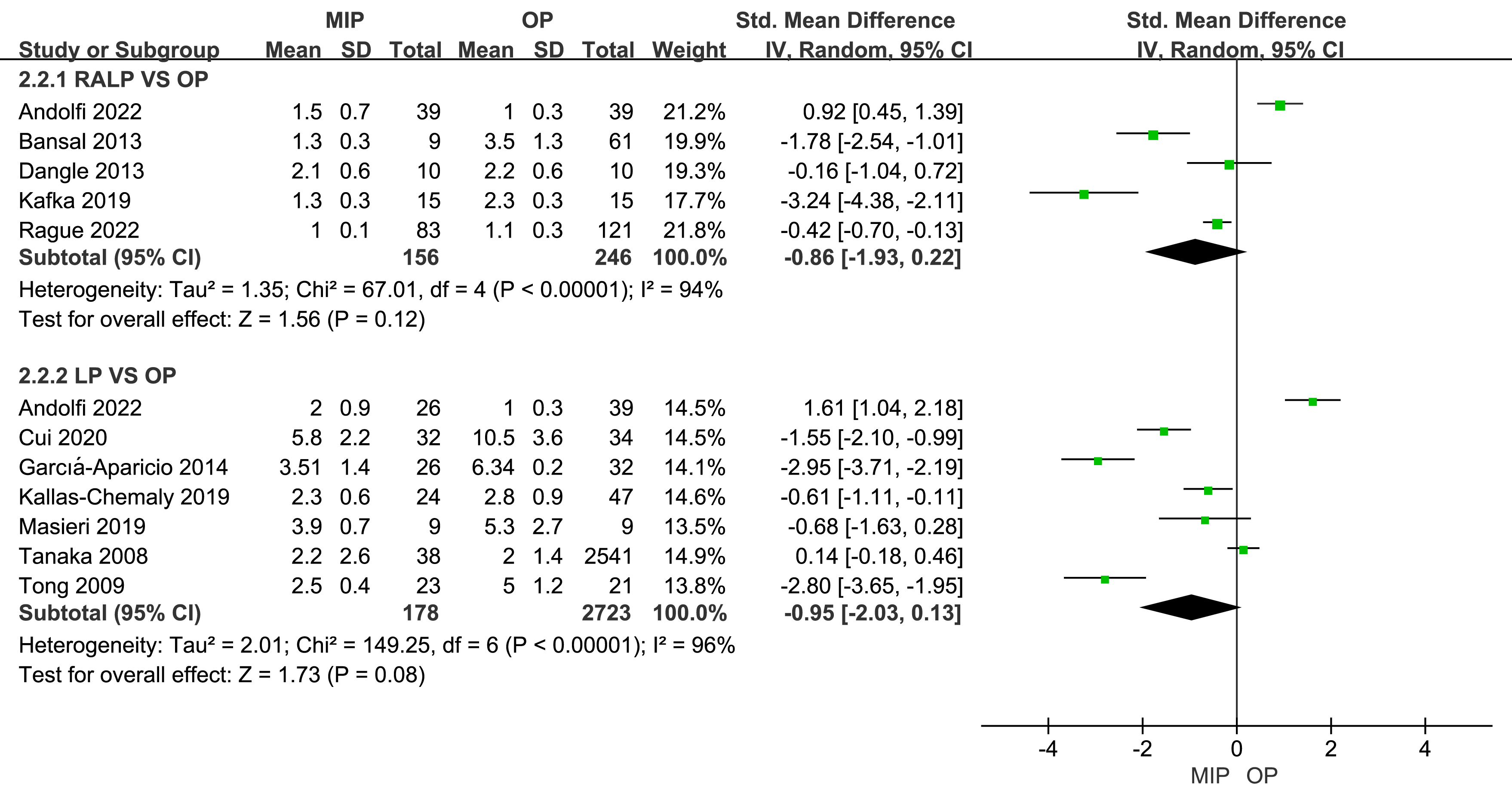

Supplement: Figure S2 [file peerj-11-16468-s005.png]

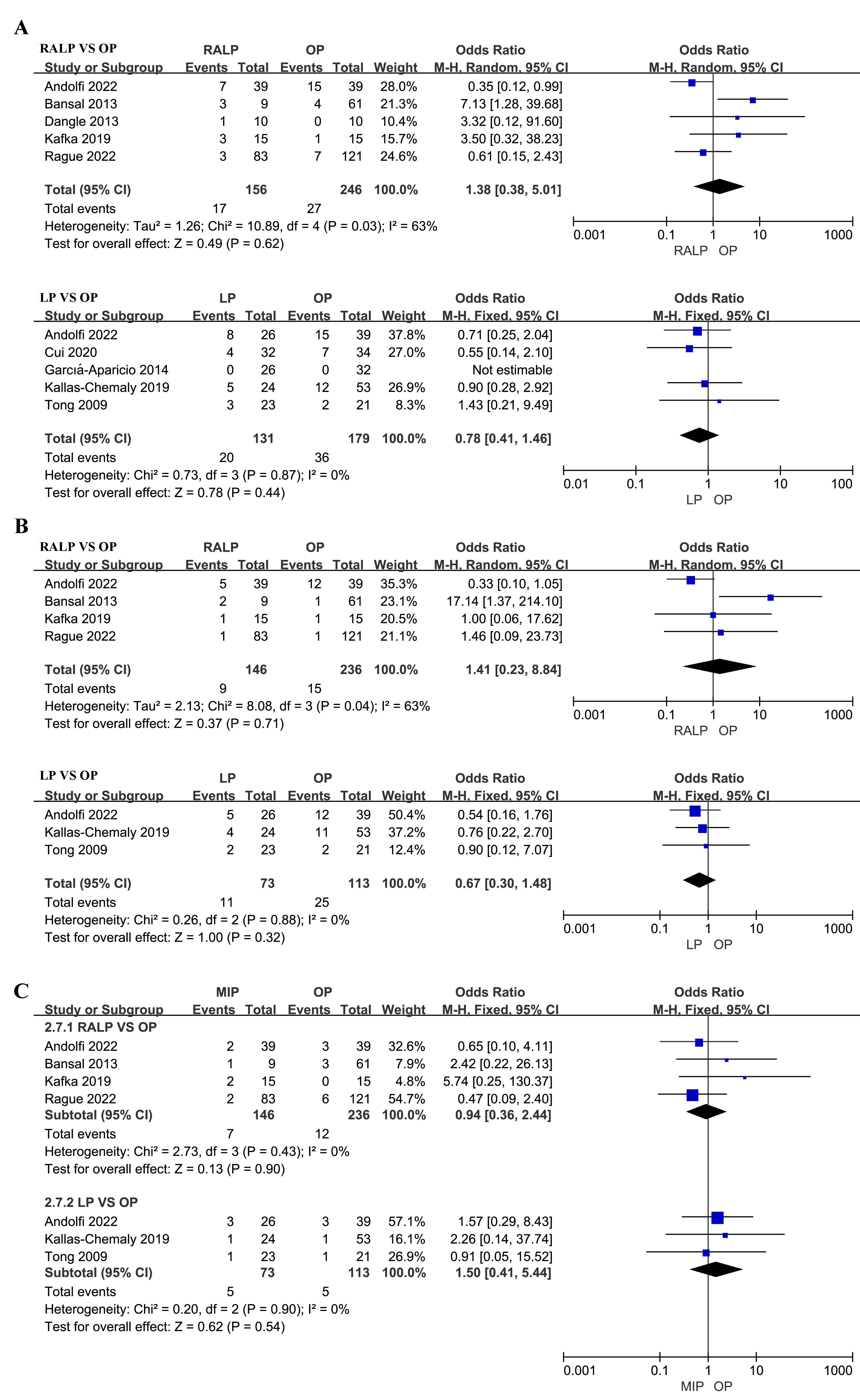

Supplement: Figure S3 [file peerj-11-16468-s006.png]

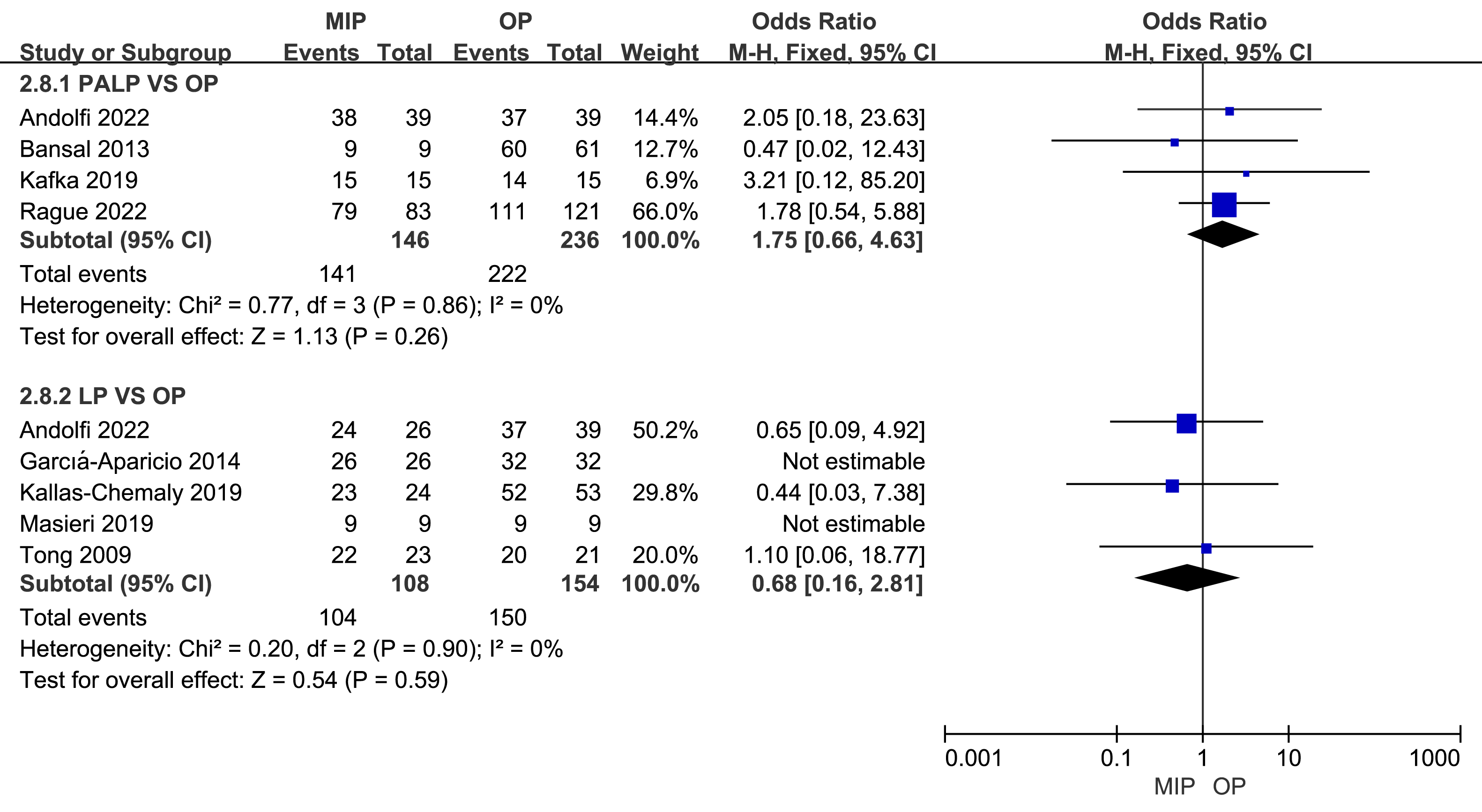

Supplement: Figure S4 [file peerj-11-16468-s007.png]
