# Supplementary material for: Minimally invasive pyeloplasty versus open pyeloplasty for ureteropelvic junction obstruction in infants: a systematic review and meta-analysis
Source: PeerJ. 2023 Nov 20;11:e16468. doi: 10.7717/peerj.16468 (PMC10666611; doi:10.7717/peerj.16468)
Supplement: Table S1 [file peerj-11-16468-s008.docx]

**For English databases**

**ID Search**

#1 ureteropelvic junction obstruction [All fields]

#2 UPJO [All fields]

#3 cakut [MeSH terms]

#4 OR #1-3

#5 pyeloplasty [All fields]

#6 nephropyeloplasty [All fields]

#7 pyeloureteroplasty[All fields]

#8 anderson-Hynes dismembered pyeloplasty [All fields]

#9 dismembered pyeloplasty

#10 OR #5-9

#11 laparoscopic [All fields]

#12 laparoscopic surgery [All fields]

#13 laparoscopic surgeries[All fields]

#14 laparoscopy [All fields]

#15 laparoscopies [All fields]

#16 peritoneoscopy [MeSH terms]

#17 peritoneoscopies [MeSH terms]

#18 laparoscopic asssisted surgery [MeSH terms]

#19 laparoscopic asssisted surgeries [MeSH terms]

#20 OR #11-19

#21 robot [All fields]

#22 robotic [All fields]

#23 robot surgery [All fields]

#24 robot surgeries [All fields]

#25 robotic surgery [All fields]

#26 robotic surgeries [All fields]

#27 robotic surgical preocedure [MeSH terms]

#28 robotic surgical preocedures [MeSH terms]

#29 robot asssisted surgery [All fields]

#30 robot asssisted surgeries [All fields]

#31 robot-asssisted surgery [MeSH terms]

#32 robot-asssisted surgeries [MeSH terms]

#33 robotic asssisted surgery [All fields]

#34 robotic asssisted surgeries [All fields]

#35 robotic-asssisted surgery [MeSH terms]

#36 robotic-asssisted surgeries [MeSH terms]

#37 OR #21-36

#38 infant [All fields]

#39 infants [All fields]

#40 OR #38-39

#41 #4 AND #10 AND (#20 OR #37) AND #40

**For Chinese databases**

**For CNKI**

主题=(‘肾盂成形术’)

AND

(主题=(‘腹腔镜’) OR 主题=(‘机器人’))

AND

(主题=(‘婴儿’) OR 主题=(‘1岁’))

**For Wangfang**

(主题词=(‘肾盂成形术’) OR 关键词=(‘肾盂成形术’))

AND

(主题词=(‘腹腔镜’) OR 主题词=(‘机器人’) OR 关键词=(‘腹腔镜’) OR 关键词=(‘机器人’))

AND

(主题词=(‘婴儿’) OR 关键词=(‘婴儿’))

**For VIP**

主题=(‘肾盂成形术’)

AND

(主题=(‘腹腔镜’) OR 主题=(‘机器人’))

AND

主题=(‘婴儿’)
