# Supplementary material for: Minimally invasive pyeloplasty versus open pyeloplasty for ureteropelvic junction obstruction in infants: a systematic review and meta-analysis
Source: PeerJ. 2023 Nov 20;11:e16468. doi: 10.7717/peerj.16468 (PMC10666611; doi:10.7717/peerj.16468)
Supplement: Table S2 [file peerj-11-16468-s009.docx]

**Sensitivity analysis for model selection effects on outcomes of meta-analysis and subgroup analyses**

|  |  | Operation Time | | | | | | LOS | | | | | |
| --- | --- | --- | --- | --- | --- | --- | --- | --- | --- | --- | --- | --- | --- |
|  |  | MIP VS OP (I^2^=92%) | | RALP VS OP (I^2^=89%) | | LP VS OP (I^2^=92%) | | MIP VS OP (I^2^=95%) | | RALP VS OP (I^2^=94%) | | LP VS OP (I^2^=96%) | |
|  |  | Fixed | Random | Fixed | Random | Fixed | Random | Fixed | Random | Fixed | Random | Fixed | Random |
| Merge Analysis | SMD (95%CI) | 0.86(0.69 to 1.03) | 0.96(0.30 to 1.62) | 0.56(0.35 to 0.78) | 0.32(-0.46 to 1.10) | 1.25(0.99 to 1.51) | 1.46(0.52 to 2.40) | -0.48(-0.64 to -0.33) | -1.12(-1.82 to -0.43) | -0.32(-0.54 to -0.11) | -0.84(-1.93 to 0.22) | -0.45(-0.65 to -0.25) | -0.95(-2.03 to 0.13) |
|  | P Value | <0.00001 | 0.004 | <0.00001 | 0.42 | <0.00001 | 0.002 | <0.00001 | 0.002 | 0.004 | 0.12 | <0.0001 | 0.08 |

MIP: minimally invasive pyeloplasty, OP: open pyeloplasty, RALP: robot-assisted laparoscopic pyeloplasty, LP: laparoscopic pyeloplasty, LOS: length of stay, I^2^: I-squared test value, SMD: standard mean difference, 95%CI: 95% confidence interval.

**Sensitivity analysis assessing the impact of small sample studies on outcomes**

| Outcomes | No. of Studies | No. of Patients | | Heterogeneity | | | Merge Analysis | |
| --- | --- | --- | --- | --- | --- | --- | --- | --- |
|  |  | MIP | OP | Chi² | I^2^（%） | P Value | SMD (95%CI) | P Value |
| Operation Time | 8 | 277 | 370 | 111.65 | 94 | <0.00001 | 0.97(0.21 to 1.73) | 0.01 |
| LOS | 9 | 315 | 2911 | 182.46 | 96 | <0.00001 | -1.28(-2.08 to -0.48) | 0.002 |

LOS: length of stay, No.: number, MIP: minimally invasive pyeloplasty, OP: open pyeloplasty, Chi²: Chi-square test value, I^2^: I-squared test value, SMD: standard mean difference, 95%CI: 95% confidence interval.

**Sensitivity analysis of mixed study designs on outcomes**

| Outcomes | No. of Studies | No. of Patients | | Heterogeneity | | | Merge Analysis | |
| --- | --- | --- | --- | --- | --- | --- | --- | --- |
|  |  | MIP | OP | Chi² | I^2^（%） | P Value | SMD (95%CI) | P Value |
| Operation Time | 9 | 281 | 374 | 95.84 | 92 | <0.00001 | 1.14(0.48 to 1.81) | 0.0007 |
| LOS | 10 | 319 | 2915 | 160.03 | 94 | <0.00001 | -0.94(-1.63 to -0.25) | 0.008 |

LOS: length of stay, No.: number, MIP: minimally invasive pyeloplasty, OP: open pyeloplasty, Chi²: Chi-square test value, I^2^: I-squared test value, SMD: standard mean difference, 95%CI: 95% confidence interval.

**Sensitivity analysis excluding high-risk studies on outcomes**

| Outcomes | No. of Studies | No. of Patients | | Heterogeneity | | | Merge Analysis | |
| --- | --- | --- | --- | --- | --- | --- | --- | --- |
|  |  | MIP | OP | Chi² | I^2^（%） | P Value | SMD (95%CI) | P Value |
| Operation Time | 9 | 287 | 328 | 88.25 | 91 | <0.00001 | 1.17(0.53 to 1.81) | 0.0003 |
| LOS | 9 | 287 | 328 | 155.69 | 95 | <0.00001 | -1.22(-2.07 to -0.36) | 0.005 |

LOS: length of stay, No.: number, MIP: minimally invasive pyeloplasty, OP: open pyeloplasty, Chi²: Chi-square test value, I^2^: I-squared test value, SMD: standard mean difference, 95%CI: 95% confidence interval.
