# Supplementary material for: Minimally invasive pyeloplasty versus open pyeloplasty for ureteropelvic junction obstruction in infants: a systematic review and meta-analysis
Source: PeerJ. 2023 Nov 20;11:e16468. doi: 10.7717/peerj.16468 (PMC10666611; doi:10.7717/peerj.16468)
Supplement: Supplemental Information 10 [file peerj-11-16468-s010.docx]

1. **The rationale for conducting the systematic review / meta-analysis;**

Despite numerous reports on the success of minimally invasive pyeloplasty (MIP) in children, this technique remains under-explored in infants due to the limited abdominal space and the challenges associated with internal suturing. With the advancement of minimally invasive surgical techniques, an increasing number of studies have reported the successful application of MIP in infants. However, there is a lack of systematic evidence, which prompted us to conduct a systematic review and meta-analysis.

1. **The contribution that it makes to knowledge in light of previously published related reports, including other meta-analyses and systematic reviews**

Although ample evidence currently supports the superiority of minimally invasive pyeloplasty (MIP) over open pyeloplasty (OP) in pediatric cases, systematic evidence supporting the use of MIP in infants is scarce. Our meta-analysis reveals longer operation times, shorter hospital stays, and lower stent placement rates associated with MIP compared to OP, with no significant differences in complications observed between the two groups. Subgroup analysis results support these findings. Thus, MIP emerges as a feasible and safe alternative to OP in infants.
